# Suppression of TRPV4 channels ameliorates anti-dipsogenic effects under hypoxia in the subfornical organ of rats

**DOI:** 10.1038/srep30168

**Published:** 2016-07-20

**Authors:** Fan Yang, Li Zhou, Dong Wang, Li-Li Yang, Guo-Rong Yuan, Qing-Yuan Huang

**Affiliations:** 1 Department of Pathophysiology and High Altitude Pathology, College of High Altitude Military Medicine, Third Military Medical University, 30 Gaotanyan, Shapingba, Chongqing, 400038,China; 2 Key Laboratory of High Altitude Medicine (Third Military Medical University), Ministry of Education, Chongqing, China; 3 Key Laboratory of High Altitude Medicine, PLA, Chongqing, China; 4 Department of pharmacy, Xinqiao Hospital & The Second Affiliated Hospital, The Third Military Medical University, Chongqing, China; 5 Department of nuclear medicine, Kunming General Hospital of Chengdu Military Area, China

## Abstract

The phenomenon of water intake reduction during the 1^st^ day of hypobaric hypoxia has been known for a long time. However, the reason for the same is yet unknown. The transient receptor potential vanilloid (TRPV) channels, including TRPV1 and TRPV4, are located in the subfornical organ (SFO). These are calcium permeable cationic channels gated by various stimuli such as cell swelling, low pH, and high temperature, and participate in anti-dipsogenic effects when activated. We aimed to explore the drinking behavior of rats and the mechanism of TRPVs under hypoxia. Chemical TRPV4 inhibitors (HC-067047 and Gadolinium) or TRPV4 knockout, but not TRPV1 inhibitor SB-705498, could restore the water intake under hypoxia. Hypoxia-mediated direct activation of TRPV4 may be the reason of anti-dipsogenic effects because the serum sodium, pH, and intracranial temperature are unaltered. Interestingly, we found that hypoxia immediately increased the intracellular Ca^2+^ concentration ([Ca^2+^]_i_) in HEK293-TRPV4 cells and primary neurons from SFO region, but not in the HEK293-TRPV1 cells. Moreover, hypoxia-induced [Ca^2+^]_i_ increase depended on the indispensable hemeoxygenase-2 (HO-2) and TRPV4. HO-2 and TRPV4 were also confirmed to form a complex in SFO neurons. These results demonstrated that SFO cells sense hypoxia and activate via the HO-2/TRPV4 multiple channels, which are associated with anti-dipsogenic effects.

Water requirement at high altitude increases due to increased water loss at lower than the ambient pressure of the water vapor^1^. However, a tendency toward thirst sensation inhibition and decreasing water intake during the 1^st^ day of exposure to hypobaric hypoxia was observed in rats^2^,^3^. The discrepancy between the intake and dissipation may lead to a disordered water and electrolyte balance in the early immigrant population. It is a natural phenomenon that humans and animals adapt to a hypoxic environment by increasing the blood oxygen content as soon as possible^4^,^5^,^6^. In early hypoxia, the hemoconcentration is primarily because of less water intake, modest decrease or even increase of urine, a gradual increase in perspiration, and blood release from blood-storage organs such as the spleen^7^,^8^. These integrated factors can manipulate the hypoxic animals to enhance the blood oxygen content in a short time^5^,^8^,^9^. Less water intake is one of the necessary measures for animals to adapt to an hypoxic environment in the early phase^10^.

Thirst sensation is derived from particular ion channels; one of them is the transient receptor potential vanilloid (TRPV) family^11^,^12^,^13^, including TRPV1 and TRPV4, localized in the specialized nuclei in the circumventricular organs (CVOs). These receptors participate in the regulation of thirst sensation^14^,^15^. Moreover, functional magnetic resonance imaging (MRI) studies reveal increasing neural activity in CVOs, wherein the organum vasculosum lamina terminalis (OVLT) and the subfornical organ (SFO) reside during hypertonicity in animals^16^ and humans^17^. SFO region lacks the blood-brain barrier because of the presence of fenestrated capillary endothelium^18^. It is directly exposed to the systemic circulation and appears to detect changes in the osmotic or hormonal composition^18^. Thus, SFO neurons theoretically may detect hematological variations and impact the TRPVs’ activities.

TRPV1 and TRPV4 are considered as osmoreceptors, including central^11^ and peripheral^19^, that exist in the brain and liver, respectively. Although TRPV4 was revealed to be activated by hypertonic stimuli in mammalian cells^20^, there are two contradictory reports. One of the studies demonstrated that hypertonicity sensing is a mechanical process requiring TRPV1, but not TRPV4^13^. The other study indicated that TRPV1 and TRPV4 channels are not the primary mechanisms by which the central nervous system responds to hypertonic stimuli and increasing thirst^21^. Although the reasons for this discrepancy are not clear, these studies indicate the diverse roles of TRPV1 and TRPV4 under different stimuli (hypoxia and hypertonicity) in the body fluid homeostasis. Clarification of the molecular mechanism responsible for hypoxia sensing in CVOs neurons is a prerequisite for our understanding of the anti-dipsogenic effects. In the present study, we explored the underlying reason of anti-dipsogenic effects under hypoxia and examined the molecular basis for HEK293-TRPV4 cells and hypoxic sensing primary neurons of SFO, which may participate in the regulation of thirst sensation.

## Results

### TRPV4 suppressants, but not TRPV1, restore water intake under hypoxia

**Figure 1:**
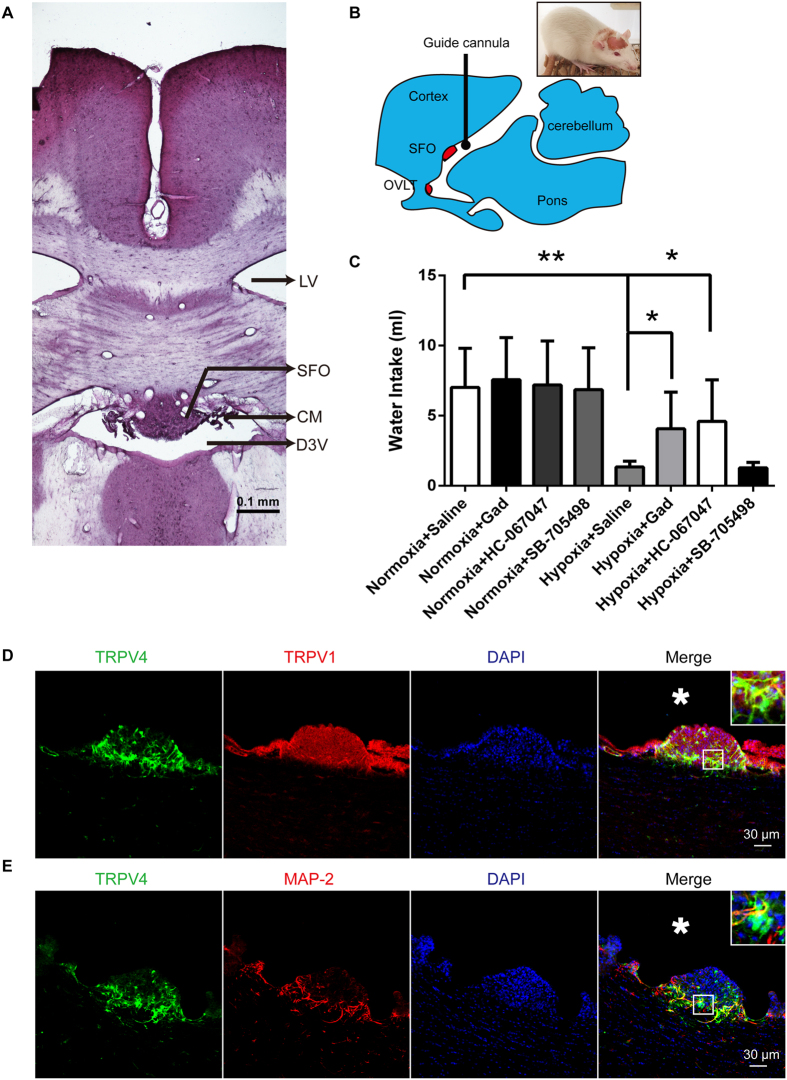
Effects of transient receptor potential vanilloid 4 (TRPV4) and TRPV1 inhibitors on the water intake of rats under hypoxia. (**A**) Hematoxylin-eosin staining showed the brain of the SFO (subfornical organ), LV (lateral ventricles), CM (choroid membranes), and D3V (third ventricle of the cerebrum). Scale bar, 0.1 mm. (**B**) Schematic showing that a guide cannula was implanted into the third ventricle and rats were recovered at 1 week after the surgery. Drugs were microinjected into the ventriculus Tertius of rats at 10 min before hypoxia. Normoxia + saline indicated the sham group. (**C**) The anti-dipsogenic effects of hypoxia (6000 m) were segmentally reversed by 1 μg TRPV4 inhibitors, gadolinium (Gad) or HC-067047, but not TRPV1 inhibitor (10 mg SB-705498) (n = 10 for each group; *P < 0.05, **P < 0.01). Immunocytochemistry examined the colocalization of TRPV4 (green) with (**D**) TRPV1 (red) and (**E**) MAP2 (red) in the SFO at 24 h after hypoxia (6000 m). Nuclei were counterstained with DAPI (blue). Scale bar, 30 μm. The rats in (**A**), (**D**), and (**E**) lack the 3^rd^ ventricular catheter.

**Figure 2:**
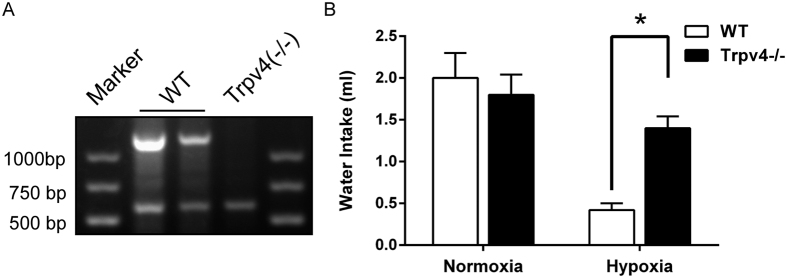
The anti-dipsogenic effects were restored in TRPV4 knockout mice under hypoxia. (**A**) A representative example of PCR products from wild-type (WT) and TRPV4−/− mice for the TRPV4 allele. The WT mice for the TRPV4 genes show two bands at 605 and 1300 bp. TRPV4 knockout mice show a single band at 605 bp. The genotype of every mouse in the current study was confirmed by PCR. (**B**) Compared with the WT, water intake was added in TRPV4 knockout mice for 6 h under hypoxia 6000 m (n = 6 for each group; *P < 0.05). No difference was observed under normoxia.

We first validated the anti-dipsogenic effects of hypoxia at simulated 6000 m altitude. The rats did not exhibit any abnormal behavior including water drinking 1 week after the surgery. Consistent with the previous findings^2^,^3^, our data showed that water intake was decreased more than 78% at 6 h as compared to the normoxia + saline group (n = 12, P < 0.01, Fig. 1C). Interestingly, we found that TRPV4 suppressants, such as gadolinium, increased the water intake by 2.7 times at 6 h as compared to the saline control group under hypoxia (n = 12, P < 0.05, Fig. 1C). Another TRPV4 inhibitor, HC-067047, was used because of low-specificity of gadolinium, and similar results were obtained. However, TRPV1 inhibitor, SB-705498, did not affect the accumulation of water intake (n = 12, Fig. 1C). To further affirm the TRPV4 channel on anti-dipsogenic effects of hypoxic, TRPV4 knockout mice was used. Although the knockout did not affect the water drinking under normal oxygen, the intake was increased 3 times at 6 h as compared to the WT under hypoxia (n = 6, P < 0.05, Fig. 2). These results indicated that TRPV4 channel was responsible for the anti-dipsogenic effect of hypoxia. In addition, immunofluorescence data showed that TRPV4 was identified by co-staining with TRPV1 (Fig. 1D) and MAP-2 (neuronal maker of a neuron) (Fig. 1E) in SFO area that is a characteristic protuberance nearby the third ventricle of the cerebrum (Fig. 1A).

### Serum sodium, pH value, and intracranial temperature were unaltered under hypoxia in rats

**Figure 3:**
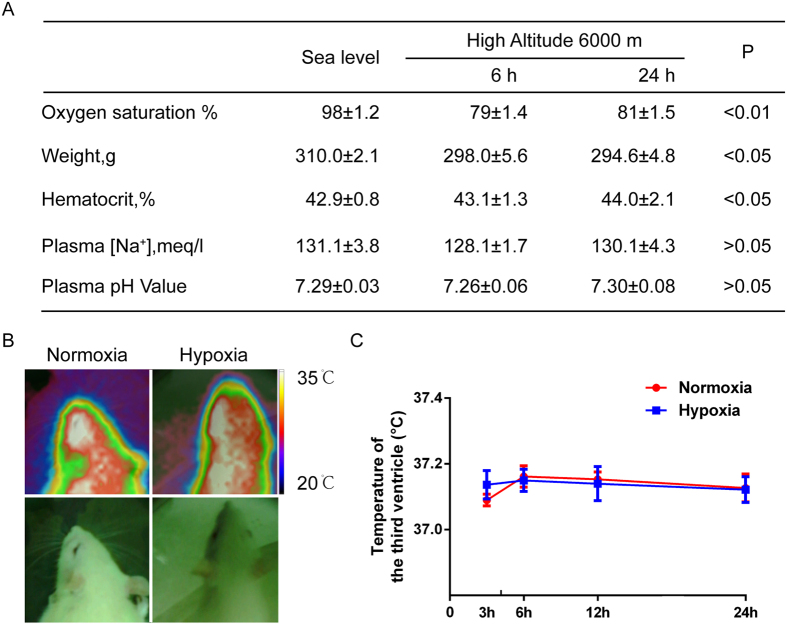
The physiological parameters during acute hypoxia 6000 m in rats. (**A**) Changes in body weight, hematocrit, serum sodium levels, and pH during the 24 h exposure to hypoxia (n = 8 for each group). (**B**) Thermal imaging of rats’ head at 24 h under hypoxia. (**C**) The temperature of the third ventricle was measured using a high accuracy thermistor thermometer.

We found that hematocrit increased after 6 and 24 h exposure to hypoxia as compared to normoxia (sea level) (n = 12, Fig. 3A). This suggests that the blood is concentrated in the early hypoxia along with less water intake. It is well-known that TRPV4 can be regulated within the microenvironment by different physical and chemical factors, such as temperature, pH, and hypertonic stimuli^9^,^22^,^23^. To explore the potential mechanism of TRPV4 in anti-dipsogenic effects under hypoxia, we detected the plasma [Na^+^], pH value, and intracranial temperature. Although oxygen saturation declined by 20% at 24 h under hypoxia (n = 12, P < 0.05, Fig. 3A), plasma [Na^+^] and pH value did not vary between normoxia and hypoxia groups (n = 12, Fig. 3A). Also, the intracranial temperature was unaltered in rats (n = 12, Fig. 3B,C). These results strongly implied that hypoxia may be involved in TRPV4 activation and reduction of water intake.

### Hypoxia-induced [Ca^2+^]_i_ increase and antagonist of TRPV4 channel inhibited this effect

**Figure 4:**
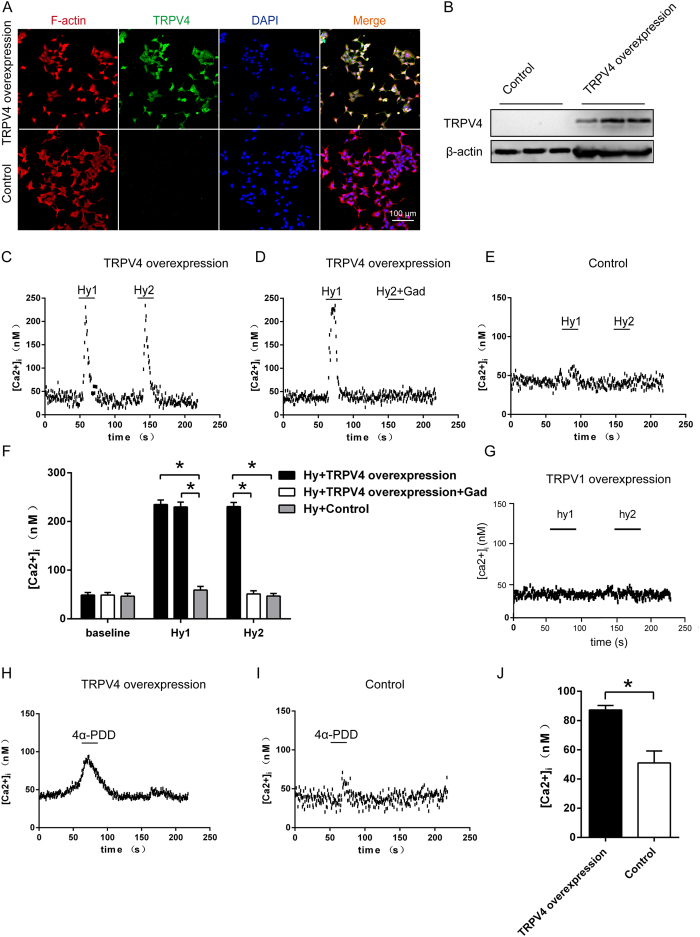
Hypoxia-induced [Ca^2+^]_i_ increase and activation by 4α-PDD (TRPV4 agonist) in TRPV4 overexpressed HEK293 cells. (**A**) Immunocytochemical staining of control and TRPV4 overexpressed cells in culture for TRPV4 (green), F-actin (red), and DAPI (blue). Scale bar, 100 μm. (**B**) Western blot for TRPV4 in the control and TRPV4 overexpressed cells. (**C**) The representative curve of [Ca^2+^]_i_ responses to two repetitive episodes of hypoxia (Hy1 and Hy2), each for 50 s in the presence of 1.5 mM extracellular Ca^2+^ in the TRPV4 overexpressed cells (n = 60). (**D**) The representative curve of [Ca^2+^]_i_ responses to repetitive episodes of hypoxia in the absence (Hy1) and presence (Hy2) of 1 μM Gad in TRPV4 overexpressed cells (n = 60). (**E**) The representative curve of [Ca^2+^]_i_ responses to repetitive episodes of hypoxia in control cells (n = 60). (**F**) The average [Ca^2+^]_i_ levels at baseline and hypoxic episodes (*P < 0.05). (**G**) The representative curve of [Ca^2+^]_i_ responses to repetitive episodes of hypoxia in TRPV1 overexpressed cells (n = 60). The representative curve of [Ca^2+^]_i_ responses to 1 μM 4α-PDD in (**H**) TRPV4 overexpressed and (**I**) control cells. (**J**) The averaged [Ca^2+^]_i_ levels of 4α-PDD treatment in TRPV4 overexpressed and control cells. The hypoxia was induced by a rapid superfusion of cells with a hypoxic medium for 2 min.

**Figure 5:**
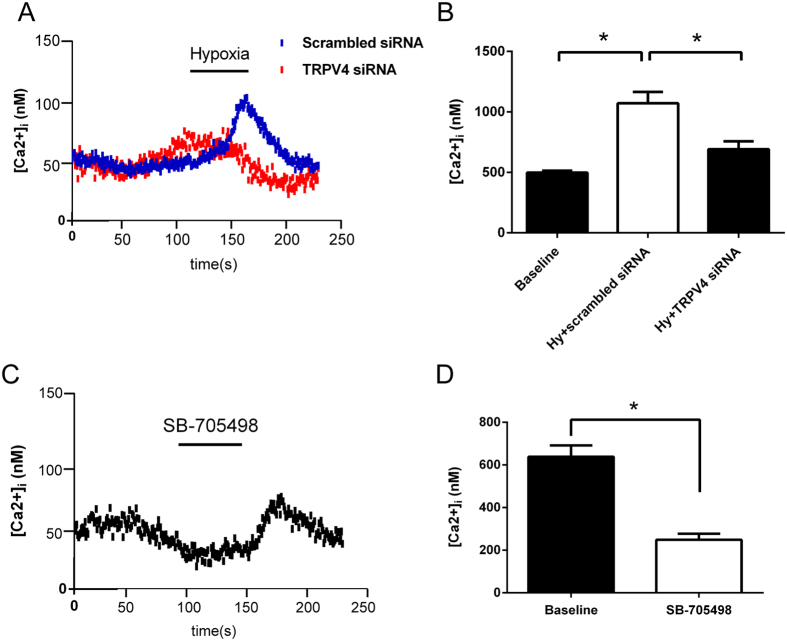
Hypoxia-induced [Ca^2+^]_i_ decreased when TRPV4 was knockdown by siRNA in primary nerve cells of SFO region. (**A**) The representative curve of [Ca^2+^]_i_ responses to hypoxia in TRPV4 siRNA and scrambled siRNA in cultured SFO neurons in the presence of 1.5 mM extracellular Ca^2+^ (n = 60). (**C**) The representative curve of [Ca^2+^]_i_ responses to 1.5 mM TRPV1 antagonist 1 μM SB-705498 in cultured SFO neurons in the presence of 1.5 mM extracellular Ca^2+^ (n = 60). Hypoxia by a rapid superfusion of cells with a hypoxic medium for 2 min. (**B**,**D**) The averaged [Ca^2+^]_i_ levels at baseline and hypoxic episodes (*P < 0.05).

To determine whether hypoxia activates the TRPV4 protein, TRPV4 overexpressed HEK293 cells were established. The protein expression was detected with the anti-V5-FITC antibody in the transfected cells, but not in the control (untransfected cells) (Fig. 4A,B). TRPV4 is a calcium channel that has been shown to be highly expressed in the cytomembrane and the perinuclear region of the transfected cells, reflecting expression in the endoplasmic reticulum,Golgi apparatus, and the membrane^22^. Moreover, extracellular Ca^2+^ rush intracellularly when activated. In the medium of constant [Ca^2+^]_o_ (1.5 mM) and temperature maintained at 37 °C by a sealed heating apparatus, hypoxia increased [Ca^2+^]_i_ in the overexpression cells (n = 60, P < 0.05, Fig. 3C), which was completely abolished or significantly inhibited by Gad (n = 60, P < 0.05, Fig. 4D). However, hypoxia could not increase [Ca^2+^]_i_ in control or TRPV1 overexpressed cells (n = 60, P < 0.05, Fig. 4E,G). 4α-PDD, an agonist of TRPV4, increased [Ca^2+^]_i_ in TRPV4 overexpressed cells (n = 60, P < 0.05, Fig. 4H), which did not occur in the control cells (n = 60, P < 0.05, Fig. 4I). We also observed the same phenomenon in primary neurons from SFO region. TRPV4 knockdown predominantly suppressed the intracellular pulse (n = 60, P < 0.05, Fig. 5A). These evidence suggest that TRPV4 calcium ion channel is immediately opened under hypoxia, and this effect within SFO is potentially associated with anti-dipsogenic effects. Moreover, TRPV1 inhibitor SB-705498 could reduce [Ca^2+^]_i_ under normoxia conditions (n = 60, P < 0.05, Fig. 5C), which implies that only a portion of TRPV1 is opened in normoxia.

### Hypoxia could not induce [Ca^2+^]_i_ increase after the HO-2 knockdown in HEK239-TRPV4 cells

**Figure 6:**
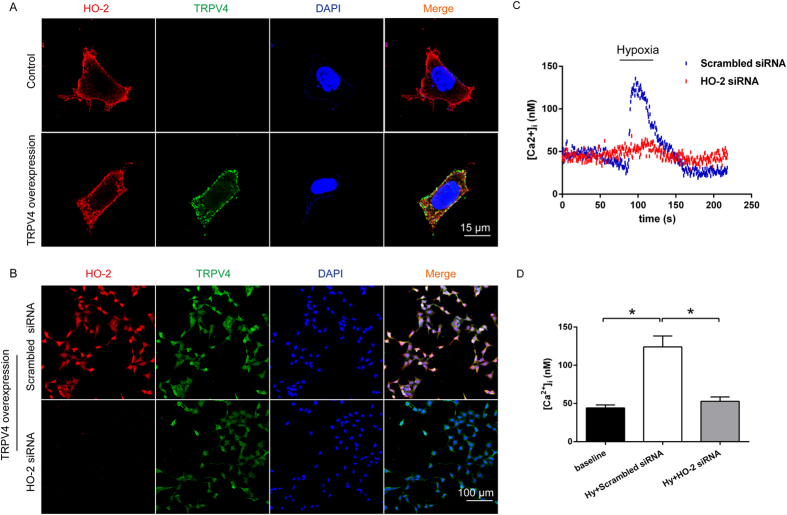
Hypoxia could not induce [Ca^2+^]_i_ increase in TRPV4 overexpressed siRNA-mediated HO-2 knockdown HEK293 cells. (**A**) The co-localization of TRPV4 (green) with HO-2 (red) in cytomembrane in TRPV4 overexpressed cells. Scale bar, 15 μm. (**B**) HO-2 was knock down by siRNA and scrambled siRNA as a control in TRPV4 overexpressed cells. DAPI (blue) as a maker of the nucleus. Scale bar, 100 μm. (**C**) The representative curve of [Ca^2+^]_i_ responses to hypoxia in HO-2 siRNA and scrambled siRNA in the presence of 1.5 mM extracellular Ca^2+^ (n = 60). Hypoxia by a rapid superfusion of cells with a hypoxic medium for 2 min. (**D**) The averaged [Ca^2+^]_i_ levels at baseline and hypoxic episodes (*P < 0.05).

To explore whether TRPV4 is an oxygen sensitive calcium ion channel, we established a HO-2 knockdown model in HEK293 cells. HO-2 is highly and constitutively expressed in neuronal and chemosensing tissues^24^,^25^, and co-localizes with the BK channel to sense oxygen concentration^26^. HO-2 is ectopically expressed in the membrane and cytoplasm of the cells and co-localizes with TRPV4 (Fig. 6A). We knocked down HO-2 by specific siRNA in HEK239-TRPV4 cells (TRPV4 overexpression) (Fig. 6B) and then performed live cell calcium imaging. Interestingly, the calcium ion pulse induced by hypoxia was barely observed during HO-2 deficiency when compared with the scrambled siRNA (n = 60, P < 0.05, Fig. 6C). These results imply that HO-2 is a precursor for hypoxia-induced TRPV4 activation.

### Hypoxia-induced [Ca^2+^]_i_ increase in an HO-2 dependent manner in primary neurons of SFO region

**Figure 7:**
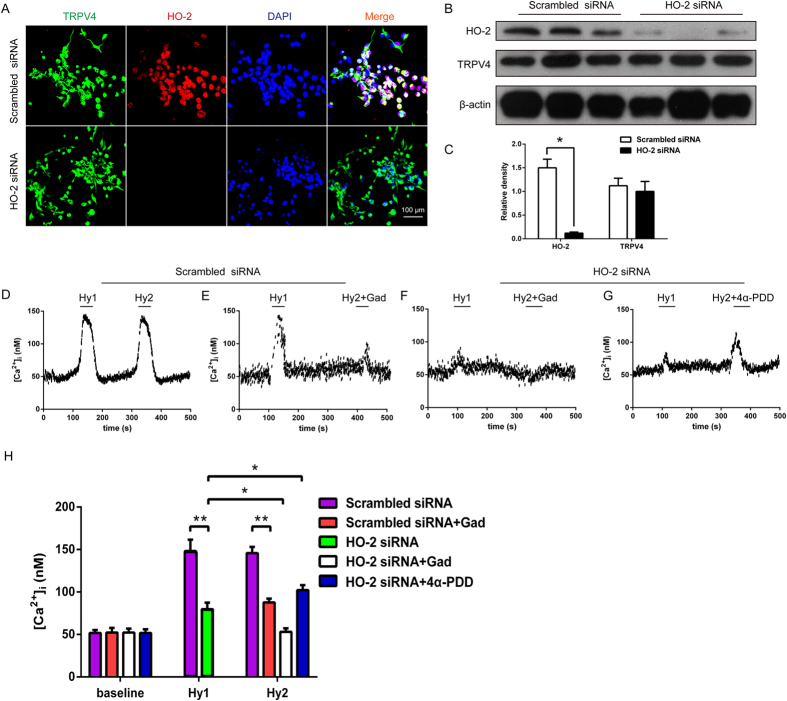
Modulation of hypoxic activation of TRPV4 channels after knockdown of HO-2 expression by siRNA in primary nerve cells of SFO region. (**A**) Co-localization of HO-2 and TRPV4 in primary nerve cells of SFO region. HO-2 was downregulated by siRNA and scrambled siRNA was used as a control. Immunocytochemical staining of cells in culture for TRPV4 (green), HO-2 (red), and DAPI (blue). Scale bar, 50 μm. (**B**,**C**) Western blot for HO-2 and TRPV4 expression. (**D**,**E**) Hypoxia induces [Ca^2+^]_i_ pulse and this is impeded by Gad in scrambled siRNA treated cells. (**D**) The representative curve of [Ca^2+^]_i_ responses to two repetitive episodes of hypoxia (Hy1 and Hy2), each for 1 min in the presence of 1.5 mM extracellular Ca^2+^ (n = 62). (**E**) The representative curve of [Ca^2+^]_i_ responses to repetitive episodes of hypoxia in the absence (Hy1) and presence (Hy2) of 1 μM Gad (n = 59). (**F**,**G**) TRPV4 modulation, but not hypoxia, can cause [Ca^2+^]_i_ pulse in HO-2 knockdown cells. (**F**) The representative curve of [Ca^2+^]_i_ responses to repetitive episodes of hypoxia in the absence (Hy1) and presence (Hy2) of 1 μM Gad (n = 59). (**G**) The representative curve of [Ca^2+^]_i_ responses to repetitive episodes of hypoxia in the absence (Hy1) and presence (Hy2) of 1 μM 4α-PDD (n = 59). Hypoxia by a rapid superfusion of neurons with a hypoxic medium for 2 min. (**H**) The averaged [Ca^2+^]_i_ levels at baseline and hypoxic episodes (**P < 0.01, *P < 0.05).

**Figure 8:**
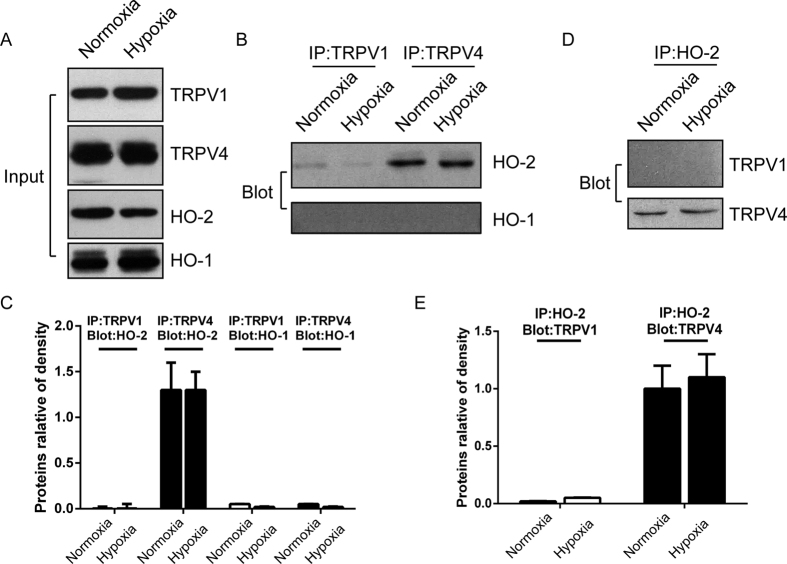
TRPV4 and HO-2 is a complex in primary nerve cells of the SFO region. (**A**) Primary SFO neuron lysate was incubated overnight with four primary antibodies (TRPV1, TRPV4, HO-2, and HO-1) for Western blot assay as control (input). (**B**) 300 μg from each group was subjected to Co-IP using anti-TRPV4 and anti-TRPV1 antibodies, and then immunoprecipitated with beads. The IP lysates (100 μg) were analyzed by Western blotting using anti-HO-2 and anti-HO-1 antibodies. (**D**) 300 μg was subjected to Co-IP using anti-HO-2. 100 μg immunoprecipitated lysates () were incubated with anti-TRPV4 and anti-TRPV1 antibodies. (**C**,**E**) Relative protein expression was calculated. All the experiments were repeated three times. Hypoxia was induced by a rapid superfusion of neurons with a hypoxic medium for 2 min.

**Figure 9:**
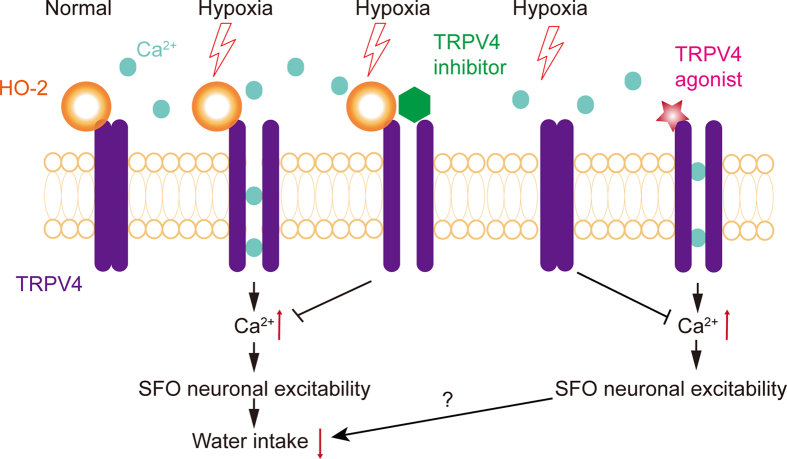
Schematic representation of the regulation of TRPV4 and anti-dipsogenic effects by hypoxia.

In an attempt to verify the synergistic effect of HO-2 and TRPV4 in the SFO region under hypoxia, primary neurons of the organ were carefully separated and treated with HO-2 siRNA. HO-2 was co-localized with TRPV4 and significantly downregulated in the primary neurons of the SFO region as detected by immunofluorescence (Fig. 7A) and Western blot (Fig. 7B). This phenomenon was consistent with the results from TRPV4-HEK293 cells. Hypoxia-induced [Ca^2+^]_i_ (n = 62, P < 0.01, Fig. 7D) was significantly inhibited by Gad in scrambled siRNA cells (n = 59, P < 0.05, Fig. 7E), and also could be restricted in HO-2 deficiency (n = 59, P < 0.05, Fig. 7D,E). Moreover, Gad (n = 59, P < 0.05, Fig. 7F) or 4α-PDD could maneuver [Ca^2+^]_i_ (n = 59, P < 0.05, Fig. 7G) in knockdown HO-2 cells, which implied that pharmacological manipulation of TRPV4 is inefficient on HO-2. We further demonstrated that HO-2 combined with TRPV4 rather than TRPV1 in SFO neuron (Fig. 8B,D) and that this complex is impregnable under hypoxic conditions. However, HO-1 does not integrate with TRPV4 or TRPV1 (Fig. 8B). Taken together, HO-2 and TRPV4 form a protein complex, which exerts a synergistic effect under hypoxia. Therefore, HO-2 is responsible for the hypoxic perception and transduction of TRPV4 signal (Fig. 9).

## Discussion

The phenomenon that cumulative water intake of the animal significantly reduced in the early stage of hypoxia, anti-dipsogenic effect, has been discovered for a long time^2^,^3^. However, the reason is yet ill-understood. In the present study, we attempted to explore the underlying mechanism and deduced three features; First, hypoxia could reduce water intake, which was irrelevant to osmotic pressure, pH, and intracranial temperature. Second, these effects are related to TRPV4 in an HO-2 dependent manner on the central nervous system. Third, the neurons in the SFO region may be responsible for these effects.

The transient receptor potential (TRP) channels, including TRPV1 and TRPV4, expressed in the neurons of the SFO area lacking blood-brain barriers, which are regarded as osmosensors in the brain^12^,^15^,^24^,^27^,^28^. To investigate the molecular mechanism of the anti-dipsogenic effects, a rat model for the third ventricular catheter was established. Interestingly, our data indicated that TRPV4 inhibitor, but not a TRPV1 inhibitor, can regain the water intake under hypoxia. Concurrently, the anti-dipsogenic effects were suppressed in TRPV4 knockout mice. This evidence strongly suggests that TRPV4 was associated with hypoxia-induced anti-dipsogenic implications in the brain. However, two complications required resolving. First, irrespective of TRPV4 inhibitor or TRPV4 knockout, anti-dipsogenic effects could not be eliminated, which indicates that other mechanisms are potentially involved. Second, both TRPV4 inhibitor and TRPV4 knockout cannot increase the water intake under normoxia, which illustrates that TRPV4 is not the key factor generating thirst perception in normoxia.

TRPVs have been reported to be activated by hypotonic stimuli, putatively by the increase of intracellular calcium ion ([Ca^2+^]_i_). However, other factors can lead to TRPVs’ activation, such as temperature and acid-base balance. TRPV4 responds to hypertonic stimuli in a temperature-dependent manner and are activated as with escalating temperatures^9^,^19^. Although the cerebrospinal fluid could not be harvested, we found that serum sodium concentrations, pH value, and intracranial temperature were not altered during hypoxia. This implied that the hypoxia-induced anti-dipsogenic effects were not secondary and that TRPV4 may be activated directly by hypoxia. In order to substantiate it, TRPV4-transfected HEK293 cells were used, which showed that [Ca^2+^]_i_ was increased by hypoxia or by a TRPV4 agonist while TRPV4 inhibitor reduced [Ca^2+^]_i_. Previous studies have revealed that TRPV4 is activated by hypoxia in a pulmonary vascular endothelial cell that is involved in hypoxic pulmonary hypertension^21^,^29^. This evidence suggested that TRPV4 is an oxygen sensitive calcium ion channel.

Therefore, it was imperative to understand how the SFO area cells containing TRPV4 change during hypoxia and achieve oxygen signal perception. Consistent with the TRPV4-HEK293 cells, we found that the SFO area primary neuron could be excited in hypoxia and inhibited by the TRPV4 antagonist. In fact, not only the partial pressure of blood oxygen (bPaO_2_) but also that of the cerebrospinal fluid (cPaO_2_) decreased when the animal remained in a hypoxic environment. The SFO area cells lacking the blood brain barrier and proximal to the third ventricle may respond to cPaO_2_ changes easily. As a result, the TRPV4 channel is opened, neurons excited and projected to the senior central cortex. However, TRPV4 channel could not sense PaO_2_ directly; we found that the activation of TRPV4 channels by hypoxia was dependent on HO-2 expression while the manipulation of TRPV4 by Gad or 4α-PDD was independent of HO-2 expression. Thus, it can be inferred that HO-2 and TRPV4 form a, ion channel complex in cytomembrane and organelles, wherein HO-2 accepts TRPV4 and react to the oxygen signal. HO-2 is an enzyme that degrades heme into biliverdin and carbon monoxide^30^,^31^. A previous study found that the knockdown of HO-2 expression reduced the calcium-sensitive potassium channel activity, and carbon monoxide, which is a product of HO-2 activity^26^. Moreover, it can be presumed that HO-2/TRPV4 complex is a key to oxygen sensing, water, and sodium balance adjustment in SFO region at high altitude.

Thirst and fluid intakes are a response to osmotic pressure hoist and body fluid deficits^11^,^32^. Though disputed, some of the brain regions and neural circuits, such as CVOs, which contains two circumventricular organs, SFO, and OVLT, participate in the physiological regulation of the fluid intake when animals exhibit body fluid deficiencies^18^,^19^. Such homeostatic regulation of fluid intake is controlled by the thirst drive that can arise when the body is dehydrated, or when the CVOs’ neurons are activated^32^,^33^. Conversely, after adequate or excess ingestion of water, inhibitory influences occur on the thirst, and fluid intake is interrupted in a regulatory manner^1^,^2^. Water requirement at high altitude theoretically increases due to increased water loss at low ambient water vapor pressure^1^. A tendency of decreased intake during ascent to higher altitudes is observed. Since the anti-dipsogenic effect is significant, it is highly possible that the reduction of water intake would help human and animal to acclimatize with the ambient environment under hypoxia. Additionally, the water intake is significantly changed in a self-regulatory manner, which leads to a decrease in plasma volumes in subjects at high altitudes, which is consistent with that reported by Surks *et al*.^6^,^10^. This improves the density of red blood cells and hypoxia tolerance but is not associated with the osmotic pressure, which is essential for maintaining the homeostatic balance^2^,^6^,^10^,^32^. Thus, our data are consistent with the hypothesis that the reduction of water intake may decrease the plasma volumes and elevate the red cell volume relatively.

On the other hand, non-brain factors may exist. We believed that the brain was a key element in the anti-dipsogenic effects. The central nervous system is sensitive to hypoxia. Hence, it is not surprising that the impairment of the neuropsychological functional occurs at high altitudes. However, a clear understanding of the effect of hypoxia on the brain remains elusive^34^,^35^. Our results only appear to scrape the tip of the iceberg while addressing the hypoxic influence on the brain function and behavior, including drinking, eating, and sleeping. The current study about water intake may provide some insights into these issues.

In summary, we show that HO-2/TRPV4 channels are involved in the increase of [Ca^2+^]_i_ signaling occurring after hypoxia in the SFO areas of adult rats. Moreover, it sheds light on the anti-dipsogenic effects and sodium water and electrolyte balance in the new migrated plateau population.

## Methods

### Animal and surgical procedure

This study was performed in accordance with China’s animal welfare legislation for the care and use of animals and approved by the Third Military Medical University Chongqing, China. Male Sprague-Dawley (SD) rats, weighing 275–300 g, were housed in specific pathogen-free (SPF) condition with free access to water and food. The rats were anesthetized with an i.p. injection of chloral hydrate (100 mg/kg body weight) and placed in a stereotaxic apparatus. A guide cannula (AG-8; Eicom, Tokyo, Japan) was implanted into the third ventricle (coordinates from bregma: 2.2 mm posterior, 0.9 mm lateral, 8.4 mm below skull surface angled at 5° vertical towards the midline)^29^ and fixed to the skull with dental cement and small screws according to the coordinates provided by Klippel’s atlas. During the 1 week postoperative recovery period, rats were acclimated to handling and the experimental cage used for drug administration and thermistor probe. A total of 93 rats were cannulated, 13 of which were removed from the study because of weight loss in three rats after the operation and because of cannula displacement in 10 rats.

### Genotyping

TRPV4 was obtained from Dr. Zha Ke-xin (Nanjin University, China). TRPV4−/− mice, were previously made by the deletion of exon 12, which encodes the pore-loop and adjacent transmembrane domains^36^. Genomic DNA was isolated from tail snips (0.5 cm) using DNeasy Blood and Tissue Kit (Qiagen, Valencia, CA, USA). 0.4 M primers were used for TRPV4 genotyping (Invitrogen, Carlsbad, CA, USA). Sense primer: 5′-CATGAAATCTGACCTCTTGTCCCC-3′ and antisense primer: 5′-TTGTGTACTGTCTGCACACCAGGC-3′). PCR reaction mixture contained Herculase buffer (Stratagene, La Jolla, CA, USA), 0.2 mM each dNTP, 2% DMSO, 1.25 units Herculase enhanced DNA polymerase, 0.6 units Platinum Taq DNA Polymerase (Invitrogen), and 100 ng of genomic DNA. PCR cycling conditions were 94 °C (3 min) followed by 35 amplification cycles (94 °C, 30 s; 68 °C, 1.5 min; 72 °C, 2 min) and 72 °C for 10 min. 15 μL PCR amplicon was analyzed on a 1.5% agarose gel containing 0.5 μg/mL ethidium bromide for 1 h at 70 V and visualized with a UV transilluminator.

### Thirst studies

All the rats were prohibited from drinking water one day before the experiment and were randomly divided into 8 groups (10 rats in each group) as follows: 1) Normoxia + saline; 2) Normoxia + 1 μg gadolinium (Sigma, St. Louis, MO, USA); 3) Normoxia + 1 μg HC-067047 (Sigma); 4) Normoxia + 10 mg SB-705498 (MedchemExpress, CA, USA); 5) Hypoxia + saline; 6) Hypoxia + 1 μg gadolinium; 7) Hypoxia + 1 μg HC-067047; 8) Hypoxia + 10 mg SB-705498. Normoxic and hypoxic animals were placed in an environment of sea level or a hypobaric chamber simulating an elevation of 6000 m for 6 h. 10 min before the experiment, 50 μL drug solutions were injected into the ventricle by using a microinjection cannula inserted into the guide cannula. The microinjection cannula was connected via a polyethylene tube to a microsyringe containing different drug solutions. The control groups that did not receive drugs were consecutively administered with sterile saline of the same volume. 5 min after the injection, the microinjection cannula was switched to a dummy cannula, and the rats were placed in the normoxic or hypoxic environment. Blood samples were withdrawn from a femoral artery of the normoxia + saline and hypoxia + saline group animals and analyzed at the appropriate time. TRPV4−/− mice and WT mice were also used to observe the water intake under normoxia (sea level) or hypoxia (6000 m) (6 rats in each group). To ensure the accuracy of measurements, gas was eliminated from the drinking water and filled in the bottles. The consumption of water was measured as (the original water weight) − (the current water weight) at 6 h after hypoxia.

### Cranial thermometry

The intracranial temperature was measured by High Accuracy Handheld Thermistor Thermometer (Omega, HH41, USA) and Precision Thermistor (Omega, ON-403-PP tubular stainless, USA) which was inserted into the third ventricle by guide cannula. This set of devices has interchangeable sensors with an accuracy up to ±0.1 °C. The thermal imaging of rats’ head was carried out with an infrared thermometer (Fluke, vt04, USA). The external environment was continuously maintained at 25 °C.

### Cell culture and overexpression

TRPV4 and TRPV1 overexpressed HEK293 cell lines were set up as described previously^22^. The cells were grown in standard Dulbecco’s modified Eagle’s medium at 37 °C, pH 7.4, containing 10% fetal bovine serum (Sigma). The pcDNA3.1/TRPV4-V5 plasmid was transiently transfected into HEK293 cells using the Lipofectamine-2000 kit (Invitrogen). The pcDNA3.1/V5-His vector containing a LacZα cDNA insert with the fused V5 epitope was regarded as a positive control while the negative control had no insert. These verified the cloning and transfection efficiencies. When transiently transfected for 48-72 h, 30–60% cells expressed the V5 epitope, as assessed by immunocytochemistry.

### SFO dissociation

Dissociated SFO neurons were prepared as previously described^37^. Briefly, male SD rats (275–300 g) were decapitated, and their brains were removed and immediately immersed in ice-cold Ca^2+,^ and Mg^2+^-free HBSS supplemented with 0.05 M sucrose. A tissue block containing the hippocampal commissure and SFO was freed from the brain, using a dissecting microscope (Olympus, SZ61), to separate the SFO from the surrounding tissue. The isolated organ was immersed in 1 mg/mL trypsin and incubated at 37 °C in 5% CO_2_ for 15 min. The cells were then suspended in ice-cold Ca^2+^ containing HBSS (Invitrogen), filtered through a 100 μm nylon cell strainer (BD Falcon, NY, USA), and centrifuged at 900 × *g* for 5 min. The supernatant was removed, cells re-suspended, and again centrifuged, following which the cells were resuspended and plated n glass dishes for imaging (MatTek, Ashland, MA, USA). The cells were further cultured in neurobasal medium (Invitrogen) at 37 °C for a minimum of 24 h before Ca^2+^ imaging and immunocytochemistry.

### siRNA transfection of cells

Cells were grown in 24-well plates to 65–70% confluency. Transfection of HO-2 siRNA (sense: 5′-GGACAUGGAGUAUUUCUUUTT-3′, antisense: 5′-AAAGAAAUACUCCAUGUCCTT-3′) or TRPV4 siRNA (sense: 5′-ACGAGACTAGTGAGACGTG-3′, antisense: 5′-CCTGCTCAACATGCTCATTG-3′) were carried out according to the manufacturer’s instructions (Santa Cruz Biotech, Dallas, TX, USA)). Briefly, the cells were incubated with a transfection solution containing a mixture of siRNA (100 nM) and siRNA transfection reagent (5 mL) for 6 h. Subsequently, the transfection solution was replaced with neurobasal medium. After an additional 24 h, the cells were harvested to measure [Ca^2+^]_i_. The cells were also consecutively transfected with scrambled siRNA (100 nM) to ensure specific gene silencing. Transfection efficiency was assessed by Western blot and immunofluorescence analysis.

### Immunofluorescence and hematoxylin-eosin staining

After 24 h of hypoxia, brain tissue was removed, fixed in 4% formaldehyde, cryoprotected in a 30% sucrose solution, sliced into 20 μm sections using Leica Microsystems Nussloch GmbH (D-69226, Germany), and stained overnight with primary antibodies at 4 °C. Primary SFO neurons and HEK293 cells were cultured on glass coverslips. The treatments’ cells were fixed with 4% paraformaldehyde solution and permeabilized using 0.1% Triton X-100. Following blocking with 10% normal goat serum, the cells were incubated with primary antibodies: anti-TRPV4 (1:200), anti-TRPV1 (1:300), anti-MAP-2 (1:300), anti-HO-2 (1:200), and anti-F-actin (1:200) (Abcam, Cambridge, UK).The samples were washed and probed with the appropriate secondary antibodies (Jackson Immunoresearch, West Grove, PA, USA). Micrographs were randomly selected and captured under a fluorescent microscope and analyzed using MagnaFire SP 2.1B software (Olympus, Melville, NY, USA). Additionally, brain sections were also stained with hematoxylin and eosin to observe the SFO region microscopically.

### Western blotting

Total protein was extracted and concentrations estimated using a bicinchoninic acid (BCA) protein assay. The following antibodies were used: anti-TRPV4 (1:1000), and anti-HO-2 (1:1000) from Abcam, and anti-β-actin (1:1000, Santa Cruz Biotechnologies). The immunoreactive bands were visualized using enhanced chemiluminescence (Amersham Biosciences, Arlington Heights, IL, USA) according to the manufacturer’s instructions. The expression of target proteins was detected on a bio-imaging system (VersaDoc MP 4000; Bio-Rad, Hercules, CA, USA) and ImageJ software analyzed the densitometric values. β-actin was used as an internal control.

### Co-immunoprecipitation assay

3 μg of primary antibody (TRPV1: 1:50; TRPV4: 1:100; HO-2: 1:100) was added to 500 μg of protein extract and incubated at 4 °C for 60 min with gentle agitation. Then, 20 μL of Protein A/G Plus-Agarose beads (Abcam) was added and incubated at 4 °C overnight followed by centrifugation at 2500 rpm for 5 min at 4 °C. The supernatant was discarded, and the Co-IP products were washed thrice with PBS. Subsequently, the precipitates were resuspended in 40 μL of sample buffer for Western blot assay: TRPV1(1:1000), TRPV4 (1:1000), HO-1(1:500), and HO-2(1:1000) were used as primary antibodies (Abcam).

### Internal flow of Ca^2+^

Internal flow of Ca^2+^ was measured in 4-(2-hydroxyethyl)-1-piperazineethanesulfonic acid-buffered saline (HBS) containing: NaCl 135 mM, KCl 5.0 mM, CaCl_2_ 1.5 mM, MgSO_4_ 1.2 mM, D-glucose 10 mM, and HEPES 5 mM (pH 7.40) using Fura-3 (Invitrogen, CA). For hypoxic stimulation, the HBS under a normoxic condition in the bath was quickly switched to hypoxic HBS through a sealed perfusion system (GM8000, Tokaihit^®^). Before the experiments, the hypoxic HBS was arranged by continuously equilibrating with 95% N_2_-5% CO_2_. HBS was maintained at 37 °C during the experiments.

### Statistical analysis

Data were analyzed by SPSS13.0 software and presented as means ± standard deviation (SD). A one-way analysis of variance (ANOVA) with repeated measures was used to estimate the significance of water intake at different times. The Student’s t-test and ANOVA were carried out for two or multiple group comparisons, respectively. Results were statistically significant at P < 0.05.

## Additional Information

**How to cite this article**: Yang, F. *et al*. Suppression of TRPV4 channels ameliorates anti-dipsogenic effects under hypoxia in the subfornical organ of rats. *Sci. Rep.* **6**, 30168; doi: 10.1038/srep30168 (2016).
